# Characterization of adverse reactions to two antihistamine drugs: A descriptive analysis from WHO-VigiAccess

**DOI:** 10.1371/journal.pone.0355852

**Published:** 2026-08-12

**Authors:** Yiheng Li, Jing Zhu, Qian Guo, Jiaman Liao, Lixia Li, Jing Hu, Xiaoliang Zhang

**Affiliations:** 1 Longgang Central Hospital, Shenzhen, China; 2 School of Integrative Medicine, Nanjing University of Chinese Medicine, Nanjing, China; 3 Department of Rhinology, The First Affiliated Hospital of Zhengzhou University, Zhengzhou, China; Satyawati College, University of Delhi, INDIA

## Abstract

**Introduction:**

Antihistamines like azelastine and levocabastine are key in treating allergies, including allergic rhinitis, conjunctivitis, and urticaria. This study analyzed adverse drug reactions linked to these drugs using data from the WHO VigiAccess database, comparing their adverse drug reaction profiles to help physicians choose lower-risk options for personalized treatment.

**Methods:**

A retrospective descriptive analysis was used, with data from WHO-VigiAccess on azelastine and levocabastine. Included were global patient demographics (age, gender, location) and details on illness systems/symptoms from direct and annual adverse drug reaction reports. Adverse drug reaction percentages for each drug were calculated for comparison.

**Results:**

Over the study period, 11,592 adverse events related to both drugs were reported. Both had high rates of General disorders and administration site conditions. Azelastine showed more Nervous system disorders. Levocabastine had higher rates of endocrine disorders, injury/poisoning/procedural complications, and Nervous system disorders. The top five adverse events were: General disorders (4463, 36.7%), Nervous system (3475, 28.6%), Gastrointestinal (1558, 12.8%), Respiratory (1515, 12.5%), and Injury/poisoning (871, 7.2%). For SOC-reported ADRs, > 10% incidence occurred in 2 azelastine cases and 4 levocabastine cases.

**Conclusion:**

Clinicians should note antihistamine ADRs. This study identifies common and specific reactions reported to the WHO, aiming to guide safer, more judicious use of these drugs.

## Introduction

**Fig 1:**
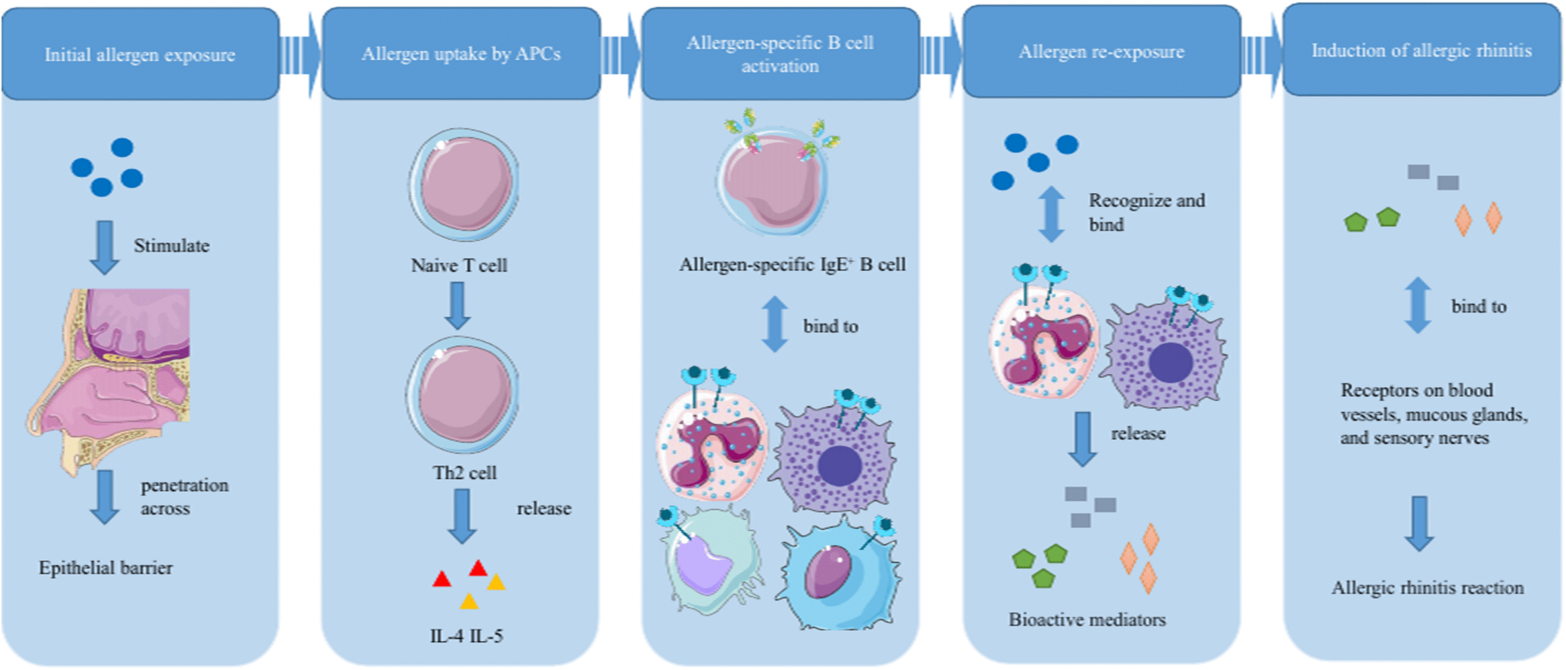
Pathogenesis of allergic rhinitis. Allergens penetrating the nasal epithelial barrier are taken up by antigen-presenting cells, which promote the differentiation of naive T cells into Th2 cells. Th2-derived cytokines drive allergen-specific IgE production by B cells. IgE binds to high-affinity receptors on mast cells and basophils. Upon allergen re-exposure, IgE cross-linking triggers mast cell degranulation and the release of mediators such as histamine, leukotrienes, and platelet-activating factor, which act on blood vessels, mucus glands, and sensory nerves to produce AR symptoms.

Allergic rhinitis (ARs) is a prevalent chronic inflammatory condition with a global prevalence of up to 40%, exhibiting a rising trend in recent years, making it a significant global public health issue [1–3]. It usually manifests as runny nose, nasal congestion, sneezing, itchy nose, itchy eyes, and itchy throat, and so on [3]. Clinically, nasal mucosal congestion, increased secretion of nasal contents, as well as pale edema of the turbinates, can be observed [4]. This condition significantly impacts the patient’s quality of life and physical and mental health [5]. ARs episodes usually exhibit a seasonal or intermittent pattern and are highly prevalent in seasons when allergens (e.g., pollen, willow, dust mites, molds, and so on) increase. During pathogenesis, allergens entering the nasal cavity stimulate the nasal mucosa, penetrating the epithelial barrier, which are then taken up and processed by antigen-presenting cells [5]. This leads to the differentiation of naïve T cells into Th2 helper T cells capable of releasing cytokines (e.g., IL-4, IL-5). This results in the generation of B cells with allergen-specific IgE that bind to high-affinity IgE receptors on mast cells, Langerhans cells, monocytes, and basophils. Upon subsequent encounters with the allergen, the associated antigenic determinant cluster (i.e., a 7–8 amino acid peptide) is recognized by the IgE molecule in the region of the specific IgE antigen-binding site (which binds to mast cells and basophils), resulting in mast cell activation and release of biologically active mediators, such as histamine, leukotrienes, and platelet-activating factor. Binding of these mediators to receptors on blood vessels, mucus-secreting glands, and sensory nerves induces physiologic responses associated with ARs symptoms [6–9] (Fig 1). Effective treatment strategies encompass the use of mast cell stabilizers, antihistamines, glucocorticoids (GCS), leukotriene receptor antagonists, and nasal decongestants [10,11]. The combination therapy most frequently employed, involving nasal GCS administration and antihistamines, is currently regarded as the most effective treatment option [12].

Histamine exerts a significant role in the pathogenesis of AR. In the early allergic immune response, elevated mast cell activity leads to the release of histamine, that binds to H1 receptors on nasal mucosal epithelial cells, mediating increases in vascular permeability and a lowering of the epithelial barrier, bronchial smooth muscle contraction, and the recruitment of inflammatory factors [12,13]. Antihistamines such as azelastine and levocabastine are capable of inhibiting histamine-mediated allergic reactions through competitive binding to the H1 receptor with histamine, thus achieving therapeutic effects [14]. Azelastine, a phthalazinone derivative, is a second‑generation antihistamine and a selective H1‑receptor antagonist. It exhibits unique pharmacological properties by inhibiting the synthesis and/or expression of various chemical mediators of allergic reactions [15], such as leukotrienes, kallikreins, cytokines, chemokines, and superoxide radicals [16,17]. Levocabastine is also a second‑generation antihistamine and a selective, long‑acting H1‑receptor antagonist administered via the nasal and ocular routes. In controlled trials, levocabastine has been shown to be both efficacious and well‑tolerated in the treatment of AR and allergic conjunctivitis [18,19]. Clinically utilized antihistamine nasal sprays have undergone numerous animal and clinical trials leading to their clinical use. While offering therapeutic benefits, side effects are inevitable. Clinical evidence indicates that first-generation antihistamines cross the blood-brain barrier and have sedative and drying effects on patients. Second-generation antihistamines generally exhibit better tolerability and have reduced sedative and drying effects. Intranasal antihistamines may result in hyperemia and desiccation of the nasal mucosa, with epistaxis and, in some cases, headache. Other drugs for ARs, such as leukotriene modulators (e.g., montelukast), have been linked to anxiety, depression, and nightmares in some patients. Cortisol drugs also commonly induce adverse effects such as nosebleeds, and in a few patients, nasal ulcers and septal ulcers [20].

While all marketed drugs are subject to rigorous clinical trials and long-term adjustments, the sample size in such experiments is inherently limited, and it cannot fully anticipate all the symptoms that may occur when the drug is used in various types of patients, nor predict interactions with other medications in patients with multiple comorbidities [21]. Thus, more study is warranted on drug safety evaluation based on big, real-world sample data. This study identified two antihistamine nasal sprays approved by the U.S. Food and Drug Administration (FDA) for the treatment of AR, including azelastine and levocabastine. A descriptive analysis of spontaneously reported adverse drug reactions (ADRs) in VigiAccess was carried out, and the reporting rates of adverse responses between the two pharmaceuticals were compared, in an effort to clarify the parallels and differences in adverse reactions between these two medications. In this study, we used disproportionate analyses ADR signals included in the Vigiaccess database.The study aimed to detect new and unexpected ADRs not described in the drug label. Given the paucity of research on the comparison of adverse reactions between these two drugs, this study aims to offer a theoretical basis for clinicians when selecting drugs to treat patients with different ARs.

## Materials and methods

### Drug sample

Table 1 presents the drug structures, main indications, and targets of action of the two drugs primarily studied in this article. Both azelastine and levocabastine are H1-receptor antagonists, effective in the treatment of ARs and allergic conjunctivitis. Azelastine has been in clinical use since 1986, predating the commercialization of levocabastine. Azelastine is primarily administered as a nasal spray for the treatment of seasonal and perennial ARs and has demonstrated efficacy in managing urticaria. Levocabastine, available in the form of nasal sprays and eye drops, is commonly employed for symptomatic relief of ARs and allergic conjunctivitis. These medications have been in clinical use for over two decades and are accessible in China. Other antihistamines, including loratadine, cetirizine, and olopatadine, are instrumental in the management of ARs.

**Table 1:** General information on two antihistamine agents.

Drug name and brand name	Main conditions	Drug target	First marketing time
Azelastine	allergic rhinitis, allergic conjunctivitis, urticaria	H1-R	1986
Levocabastine	allergic rhinitis, allergic conjunctivitis	H1-R	1993

### Date sources

All data used in the article was obtained from the WHO-VigiAccess website (https://www.vigiaccess.org). We obtained all adverse events (AEs) that occurred after using each medication as of August 31, 2024, using the brand and generic names of the medication. The WHO information system gathers information on age group, sex, reporting year, and the world’s major continents. Excel 2016 was used to examine descriptive data.

The PIDM database is accessible through the free site WHO-VigiAccess. Users can access drug safety reports that the UMC has received. The definitions are based on the Medical Dictionary of Regulatory Activities (MedDRA) system organ categories (SOCs) and preferred terms (PTs). Therefore, a search was conducted of the records for each antihistamine, and individual AEs were identified employing the MedDRA SOC and PT criteria to examine the range of toxicities. A number of dictionaries, including the World Health Organization Adverse Reaction Terminology (WHO-ART), are the source of words used in MedDRA. According to MedDRA, there are 20 SOCs, and a thorough analysis was conducted of those directly related to disease symptoms. This study concentrated on PTs, which were aggregated data publicly available from the VigiBase database through WHO-VigiAccess.

The spontaneous reporting system, introduced in the 1960s, remains the backbone of pharmacovigilance [22]. The primary goal of spontaneous reporting is the early identification of AEs that were previously undetected. Furthermore, spontaneous reporting facilitates the acquisition of new insights into known drug-adverse reaction associations. Global safety data is compiled by the Uppsala Monitoring Centre (UMC) on behalf of the WHO’s International Drug Monitoring (PIDM). Through VigiBase, the global voluntary reporting program, UMC has gathered and preserved over 20 million ADR reports from over 170 countries by December 2018 [23]. Since 2015, public access to the data stored in VigiBase has been provided through VigiAccess. VigiAccess databases support searches by the trade name of a drug product, which then identifies the active ingredient(s) contained and displays the ADR report results based on these active ingredient(s).

### Statistical analysis

This study utilized a retrospective quantitative research design. Descriptive analysis using Excel was conducted to analyze the characteristics of ADR profiles for two drugs. The number of ADR symptoms divided by the total number of ADR complaints was used to get the ADR reporting rate for each medication. Based on the top 20 symptoms with the greatest rates of ADR reporting, common ADRs for each medicine were determined. For the purpose of a descriptive comparison, the incidence of ADR symptoms recorded for each drug was computed and examined. Descriptive variables were organized using frequencies and percentages.

Adverse event data were retrieved from the WHO-VigiAccess database. Using Python 3.10 with the requests library, the BASENAMEs of all drugs in the WHODRUG dictionary were queried, and the JSON data returned from the front-end pages were collected. The data were then structured and visualized using the Pandas library and exported to Excel spreadsheets. All subsequent disproportionality analyses, including the calculation of ROR, PRR, and their 95% confidence intervals, were performed using SAS 9.4 software.

The individual case safety reports (ICSRs) retrieved from VigiAccess were further screened according to the following criteria. Only ICSRs in which azelastine or levocabastine was recorded as a “suspected” drug were included in the analysis. Reports where these antihistamines were coded exclusively as “concomitant” medications were excluded. To ensure data quality, duplicate reports, identified by matching report identifiers, and reports with missing or incomplete adverse event information were excluded. After applying these criteria, all eligible ICSRs were retained for disproportionality analysis.

### Disproportionality analysis

We used the Reporting Odds Ratio (ROR) and the Proportional Reporting Ratio (PRR) as two disproportionality reporting methodologies based on the disproportionality analysis. A common technique in pharmacovigilance, the measure of odds imbalance serves as the foundation for the computation of ROR and PRR. ROR quantifies the odds imbalance of reporting AEs for a specific drug relative to other drugs and is defined by the following equation:


$$\begin{array}{c} ROR=\frac{\text{a}\times \text{d}}{\text{b}\times \text{c}} \end{array}$$

where (a) is the number of reports of a specific drug and a specific AE, (b) is the number of reports of the same drug with other AEs, (c) is the number of reports of other drugs with the same AE, and (d)is the number of reports of other drugs with other AEs. The statistical robustness of the ROR calculation is dependent upon the presence of at least 5 cases (a ≥ 5) of a particular drug and adverse event combination.

PRR is an additional metric used to quantify the disproportionality of AE reports and is calculated using the following formula:


$$\begin{array}{c} PRR=\;\frac{\text{a}(\text{c}+\text{d})}{\text{c}(\text{a}+\text{b})} \end{array}$$

and like ROR, requires at least 5 cases (a ≥ 5) of a specific drug and AE combination to be considered valid.

The signal is deemed disproportionate and may raise safety concerns if the ROR value is more than two (ROR > 2) and the lower limit of the 95% confidence interval (CI) for the ROR is greater than one (lower limit of the 95% CI for ROR > 1). By using these criteria, it is ensured that random variation cannot be the cause of the observed disproportionality. The calculation formulas, standard errors, 95% confidence intervals, and signal detection criteria are summarized in Table 2.

**Table 2:** The principles of disproportionate measurement and the criteria for signal detection.

Method	Calculation formula	Criteria
ROR	$\text{ROR}=\frac{\text{a }/\text{ c}}{b\;/\;d}$	a ≥ 3 95%CI (lower limit) > 1
	$SE(lnROR)=\sqrt{\frac{\text{1}}{\text{a}}+\frac{\text{1}}{\text{b}}+\frac{\text{1}}{\text{c}}+\frac{\text{1}}{\text{d}}}$	
	$\text{9}\text{5}\%CI=\;{\text{e}}^{\text{ln}(ROR)\pm \text{1}.\text{9}\text{6}se}$	
PRR	$\text{PRR}=\frac{\text{a }/\;(\text{a}+\text{b})}{c\;/\;(c+d)}$	a ≥ 3 95%CI (lower limit) > 1
	$SE(lnPRR)=\sqrt{\frac{\text{1}}{\text{a}}-\frac{\text{1}}{\text{a}+\text{b}}+\frac{\text{1}}{\text{c}}-\frac{\text{1}}{\text{c}+\text{d}}}$	
	$\text{9}\text{5}\%CI=\;{\text{e}}^{\text{ln}(PRR)\pm \text{1}.\text{9}\text{6}se}$	
	$\chi \text{2}\;=\frac{\;{\left(\text{ad}-\text{bc}\right)}^{\text{2}}(\text{a}+\text{b}+\text{c}+\text{d})}{\left(\text{ a}+\text{b})(\text{a}+\text{c})(\text{c}+\text{d})(\text{b}+\text{d}\right)}$	a ≥ 3 PRR ≥ 2 χ2≥4

In our analysis, the application of ROR and PRR permitted a systematic assessment of the disproportionality of adverse reactions reported with antihistamines. The analysis’s findings support efforts in pharmacovigilance that try to improve drug safety.

For the targeted analysis of neurological disorders, all AEs coded to PTs belonging to the MedDRA SOC “Nervous system disorders” were included. The specific PTs identified for azelastine and levocabastine in this SOC comprised: burning sensation, hypoaesthesia, ageusia, migraine, paraesthesia, balance disorder, headache, parosmia, tremor, sedation, dysgeusia, dizziness, anosmia, and somnolence. No further restriction at the High-Level Term (HLT) or High-Level Group Term (HLGT) level was applied.

In all disproportionality analyses, the reference group for each drug–ADR pair consisted of all other drug–ADR combinations in the WHO-VigiAccess database. Specifically, the 2 × 2 contingency table was constructed with the study drug and target ADR as the index pair (cells *a* and *b*), while all other drugs and all other ADRs in the database served as the background comparator (cells *c* and *d*), as defined in the formulas above.

### Ethics statement

Ethical approval was not required for the study involving humans in accordance with the local legislation and institutional requirements. Written informed consent to participate in this study was not required from the participants or the participants’ legal guardians/next of kin in accordance with the national legislation and the institutional requirements.

## Results

### General characteristics of the cases studied

Adverse reaction reports for the drugs azelastine and levocabastine were first received in the WHO-VigiAccess database in 1988 and 1992, respectively. As of 2024, the WHO database contained 10, 793 and 799 ADR reports for these two drugs, totaling 11, 592. The numbers of AEs covered in these ADR reports were 15,330 for azelastine and 1,292 for levocabastine. Table 3 displays, among the 11, 592 reports of these two antihistamines, excluding 972 cases of unknown gender, the number of ADRs reported in females (7, 262, 62.65%) was higher than that of males (3, 376, 29.12%), with a male-to-female ratio of 2.15:1 and a gender difference. After exclusion of reports without age information, the most frequently reported age group for AEs was 45–65 years for azelastine, compared to 18–44 years for levocabastine. Over half of the reported cases of azelastine occurred in the Americas, and close to 90% of the cases of levocabastine occurred in Europe. Table 3 also lists the year of reporting for each study drug. Both drugs were documented through 2010. Over the past decade, azelastine exhibited the highest ADR incidence in 2020 and was also higher in 2016 and 2023 than in other years; levocabastine exhibited a higher ADR incidence in 2022 than in other years. ADR incidence rates for both drugs have shown an increasing trend over the past decade, excluding years with significant increases.

**Table 3:** Characteristics of ADR reports of two antihistamines.

	Azelastine	Levocabastine
**Number of ADR reports**	10793	799
**Female**	6820 (63.2%)	442 (55.3%)
**Male**	3160 (29.3%)	216 (27.0%)
**Unknown**	813 (7.5%)	141 (17.6%)
**0 - 27 days**	15 (0.1%)	1 (0.1%)
**28 days to 23 months**	5 (0.1%)	3 (0.4%)
**2 - 11 years**	131 (1.2%)	55 (6.9%)
**12 - 17 years**	134 (1.2%)	28 (3.5%)
**18 - 44 years**	1736 (16.1%)	199 (24.9%)
**45 - 64 years**	2383 (22.1%)	140 (17.5%)
**65 - 74 years**	1147 (10.6%)	47 (5.9%)
**≥ 75 years**	785 (7.3%)	38 (4.8%)
**Unknown**	4457 (41.3%)	288 (36.0%)
**Africa**	4 (0.0%)	6 (0.8%)
**Americas**	5606 (51.9%)	26 (3.3%)
**Asia**	3665 (34.0%)	34 (4.3%)
**Europe**	1484 (13.7%)	704 (88.1%)
**Oceania**	34 (0.3%)	29 (3.6%)
**2024**	883 (8.2%)	31 (3.9%)
**2023**	1239 (11.5%)	59 (7.4%)
**2022**	371 (3.4%)	149 (18.6%)
**2021**	503 (4.7%)	40 (5.0%)
**2020**	2385 (22.1%)	50 (6.3%)
**2019**	744 (6.9%)	40 (5.0%)
**2018**	663 (6.1%)	47 (5.9%)
**2017**	537 (5.0%)	17 (2.1%)
**2016**	1188 (11.0%)	38 (4.8%)
**2015**	449 (4.2%)	26 (3.3%)
**Before 2014**	1831 (17.0%)	302 (37.8%)

### Distribution of 27 SOCs for 2 antihistamines

Table 4 displays the rate of 27 SOC reports for the 2 antihistamines. The highest rates of adverse reactions observed in both azelastine and levocabastine were General disorders and administration site conditions. Furthermore, azelastine treatment exhibited a higher incidence of adverse reactions for Nervous system disorders. Levocabastine treatment also exhibited higher rates of endocrine disorders, AEs related to injury, poisoning, and procedural complications, and Nervous system disorders were also higher than in other groups.

**Table 4:** ADR number and report rate of 27 SOCs of two antihistamines.

	Azelastine (N = 15330)	Levocabastine (N = 1292)
**Blood and lymphatic system disorders**	17 (0.11%)	2 (0.15%)
**Cardiac disorders**	180 (1.17%)	24 (1.86%)
**Congenital familial and genetic disorders**	7 (0.05%)	1 (0.08%)
**Ear and labyrinth disorders**	85 (0.55%)	7 (0.54%)
**Endocrine disorders**	4 (0.03%)	261 (20.20%)
**Eye disorders**	801 (5.23%)	–
**Gastrointestinal disorders**	1506 (9.82%)	52 (4.02%)
**General disorders and administration site conditions**	4183 (27.29%)	280 (21.67%)
**Hepatobiliary disorders**	16 (0.10%)	1 (0.08%)
**Immune system disorders**	219 (1.43%)	30 (2.32%)
**Infections and infestations**	268 (1.75%)	56 (4.33%)
**Injury poisoning and procedural complications**	739 (4.82%)	132 (10.22%)
**Investigations**	270 (1.76%)	11 (0.85%)
**Metabolism and nutrition disorders**	116 (0.76%)	3 (0.23%)
**Musculoskeletal and connective tissue disorders**	152 (0.99%)	13 (1.01%)
**Neoplasms benign malignant and unspecified incl cysts and polyps**	17 (0.11%)	1 (0.08%)
**Nervous system disorders**	3340 (21.79%)	135 (10.45%)
**Pregnancy puerperium and perinatal conditions**	9 (0.06%)	3 (0.23%)
**Product issues**	649 (4.23%)	32 (2.48%)
**Psychiatric disorders**	441 (2.88%)	37 (2.86%)
**Renal and urinary disorders**	108 (0.70%)	4 (0.31%)
**Reproductive system and breast disorders**	39 (0.25%)	–
**Respiratory thoracic and mediastinal disorders**	1399 (9.13%)	116 (8.98%)
**Skin and subcutaneous tissue disorders**	582 (3.80%)	71 (5.50%)
**Social circumstances**	27 (0.18%)	3 (0.23%)
**Surgical and medical procedures**	28 (0.18%)	5 (0.39%)
**Vascular disorders**	128 (0.83%)	12 (0.93%)

The five most common AEs included General disorders and administration site conditions (4463, 36.7%), Nervous system disorders (3475, 28.6%), Gastrointestinal disorders (1558, 12.8%), Respiratory, thoracic, and mediastinal disorders (1515, 12.5%), and Injury, poisoning, and procedural complications (871, 7.2%). Of the SOC-reported ADRs, the incidence of more than 10% occurred in 2 cases of azelastine and 4 cases of levocabastine.

### Disproportionation analysis based on neurological disorders

By observing and comparing the SOC distribution of the two antihistamines, our analysis revealed that neurological disorders were the most frequently reported AEs. To further compare the two drugs, we conducted a signal disambiguation analysis focusing on neurological disorders. We employed the ROR and PRR methods. Table 5 presents the results of the signal disambiguation analysis, revealing the following ROR values for the two drugs: azelastine: 2.48 (2.10–2.92); levocabastine: 0.40 (0.34–0.48). The results suggest that azelastine is associated with a higher likelihood of neurologic disorders compared to levocabastine.

**Table 5:** Disproportionation analysis based on neurological disorders.

	ROR (95%CI)	PRR (95%CI)
**Azelastine**	2.48 (2.10-2.92)	2.16 (2.10-2.93)
**Levocabastine**	0.40 (0.34-0.48)	0.46 (0.34-0.48)

### The most common adverse effects of the two antihistamines

Table 6 lists the 10 most frequently reported ADRs for the two medications, expressed as preferred terminology from the SOC. Common adverse reactions for both antihistamines included drowsiness, drug inefficacy, dizziness, eye irritation, headache, nausea, epistaxis, fatigue, dyspnea, and pruritus. Adverse reactions such as treatment failure, taste reversal, and dry mouth were particularly associated with azelastine, whereas adverse reactions such as unexpected treatment response, eye pain, and ocular congestion were particularly associated with levocabastine.

**Table 6:** Top 10 ADRs of antihistamines.

Azelastine		Levocabastine	
ADR	Report rate	ADR	Report rate
**Treatment failure**	9.81%	Therapeutic response unexpected	4.91%
**Somnolence**	7.37%	Off label use	4.35%
**Dysgeusia**	5.05%	Eye irritation	4.29%
**Drug ineffective**	4.69%	Headache	3.48%
**Dizziness**	2.62%	Fatigue	3.42%
**Eye irritation**	1.99%	Eye pain	3.04%
**Dry mouth**	1.91%	Epistaxis	2.80%
**Headache**	1.83%	Ocular hyperaemia	2.11%
**Nausea**	1.40%	Vision blurred	2.05%
**Nasal discomfort**	1.28%	Drug ineffective	1.80%

### Serious adverse reactions to two antihistamines

The WHO-VigiAccess allowed us to identify the major AEs, including life-threatening events, disability, and congenital malformations, for the two main antihistamines studied in this paper. The incidence of serious AEs was 0.07% for azelastine and 0.08% for levocabastine. Although most of the adverse effects are mild and related to the individual patient’s constitution, there are some serious adverse effects such as hypertensive crisis and congenital malformations that require hospitalization or may even lead to patient death. No serious adverse reactions involving disability or congenital malformations have been identified with levocabastine.

### Similarities and differences in common adverse effects of these two antihistamines

By comparing the top 10 ADRs reported in the SOC for both antihistamines, a total of 130 identical signals were found in the PTs. All common signals were categorized and presented in S1 Table. The SOC with the highest number of ADR signals was Respiratory, thoracic, and mediastinal disorders. The top five identical ADR signals were respiratory, thoracic, and mediastinal disorders, eye disorders, general disorders and administration site conditions, nervous system disorders, and gastrointestinal disorders. The two drugs also each had different adverse reactions (see S2 Table), which are shown in the charts and are not described here. As can be seen from the figure, azelastine has unique adverse effects in gastrointestinal disorders, general disorders and administration site conditions, injury, poisoning, and procedural complications, and product issues, while levocabastine has unique adverse effects in immune system disorders, infections and infestations, injury, poisoning, and procedural complications, psychiatric disorders, skin and subcutaneous tissue disorders, and other unique adverse reactions.

## Discussion

This study, based on the WHO-VigiAccess database, analyzed the post-marketing adverse event reporting characteristics of azelastine and levocabastine. By 2024, a total of 11,592 reports had been included (10,793 for azelastine and 799 for levocabastine), involving 15,330 and 1,292 AEs, respectively. The proportion of female reports was significantly higher than that of male reports (62.65% vs. 29.12%). The peak age group was 45–65 years for azelastine and 18–44 years for levocabastine. Geographically, reports for azelastine mainly originated from the Americas, whereas nearly 90% of reports for levocabastine came from Europe. The most common adverse reactions overall were general disorders and administration site conditions, followed by nervous system disorders. Frequently reported adverse reactions shared by both drugs included somnolence, dizziness, headache, nausea, fatigue, dyspnoea, and epistaxis. The incidence of serious adverse reactions was less than 0.1% for both drugs, and no reports of disability or congenital malformation were identified for levocabastine. Signal comparison identified 130 shared preferred term (PT) signals, which predominantly involved the respiratory, nervous system, and general disorders system organ classes. Meanwhile, the two drugs exhibited unique adverse reaction profiles in areas such as endocrine disorders and eye disorders.

According to the data analysis, the incidence of adverse reactions in both azelastine and levocabastine was significantly higher in females than in males, with a significant gender difference. Studies have confirmed that allergic responses vary significantly throughout a woman’s reproductive life [24,25]. and that female sex hormones such as estrogen and progesterone enhance immune function, whereas male sex hormones such as testosterone suppress immune function, leading to a higher lifetime incidence of allergic disease in women than in men [26]. In addition, data analysis from Denmark has shown that the rate of ARs sensitization in women is increasing yearly in recent years, which may be one of the reasons for the high incidence of adverse reactions in women [27]. Azelastine and levocabastine have the highest concentration of adverse reactions in the age range of 18–64 years, likely because adults have a well-developed and fully functioning immune system and are more susceptible to ARs due to their stronger immune function compared to minors and the elderly [28]. This suggests that the exact correlation needs to be further investigated. Geographically, differences in the incidence of adverse reactions between the two drugs were observed; for azelastine, half of the adverse reaction instances originated in the Americas, whereas nearly 90% of the adverse reaction instances for levocabastine originated in Europe. This may be closely related to the prescribing habits of physicians in different countries. At the same time, we cannot rule out the regional influence caused by differences in population size across continents and variations in awareness of spontaneous adverse event reporting among healthcare institutions. For example, the low adverse reaction reporting rates in Africa and Oceania are associated with low drug utilization and differences in healthcare professionals’ knowledge of the drugs, a situation further exacerbated by the high cost of many drugs and complex procurement procedures [29]. No geographical studies on ARs are available now. Apart from years with notably higher data, both azelastine and levocabastine showed a yearly increase in the incidence of adverse reactions, which, in addition to the yearly increase in sensitization rates among adults, may be related to the new coronavirus in late 2019. Studies have shown that the likelihood of developing anaphylaxis after COVID-19 infection is greatly increased [30,31]. The elevated incidence of allergic disease has led to increased utilization of both drugs, which may be one reason for the continued increase in reported instances of adverse reactions.

Comparison of the adverse reaction signals for the two drugs revealed that both azelastine and levocabastine exhibited the characteristic side effects of antihistamines, namely sedation and drying effects, and the sedative effect of azelastine is more pronounced when taken with alcohol [32]. Respiratory symptoms were particularly prominent; nasal discomfort, sore throat, wheezing, nasal dryness, and dyspnea were common adverse reactions shared by both drugs. In addition, we observed that the incidence of nervous system adverse reactions exceeded 10% for both drugs, and nervous system adverse reactions accounted for the highest proportion among the adverse reaction reports for azelastine. Studies have shown that azelastine has a certain ability to cross the blood–brain barrier and, under specific experimental conditions, can induce cytotoxic effects and inhibit the proliferation of glioblastoma cell lines [33]. Furthermore, it was observed that the incidence of neurologic adverse reactions exceeded 10% for both drugs. Neurologic adverse reactions common to both drugs encompass loss of taste, loss of smell, balance disorders, headache, olfactory inversion, tremor, sedation, vertigo, and somnolence. Moreover, azelastine has been associated with adverse reactions such as seizures, loss of consciousness, and hypotonia [15,34]. Experimental evidence from canine studies has demonstrated that azelastine can induce constitutional generalized seizures, potentially elucidating the mechanism behind seizure-related adverse reactions [35]. Extensive research on azelastine indicates that it is commonly associated with sedative effects post-administration. Levocabastine is also linked to distinct neurologic adverse reactions, such as attention deficit, brain fog, mucous membrane burning sensation, dystonia, and facial palsy. Levocabastine functions as both an H1 receptor antagonist and an antagonist of the neurohypocretin NTS2 receptor [36]. Neurohypocretin is an endogenous neuropeptide that binds to the NTS2 receptor and promotes neuronal calcium inward flow, thereby participating in cellular signaling. In the central nervous system, levocabastine can act as an NTS2 receptor antagonist, thereby inhibiting NO synthase activity and regulating mitochondrial function [37]. The use of levocabastine also has an effect on Na-K-ATPase activity [38]. These processes play an important role in neural nociceptive signaling. It is plausible to infer that adverse effects such as headaches experienced by patients are associated with NTS2 signaling pathways. We also observed in the data that adverse reactions related to endocrine disorders accounted for the largest proportion of reports for levocabastine, exceeding 20% of the total AEs. This is closely linked to the role of the neuropeptide neurotensin (NT). A study on the effects of NT showed that levocabastine, acting as an NTSR agonist, exhibited a protective effect on pancreatic cells similar to that of NT and may participate in regulating certain endocrine pancreatic functions [39].

Although the present study focused on intranasal antihistamines, the AEs analyzed underscore the importance of limiting these drugs to their approved indications. Indiscriminate use of these agents in non-allergic conditions such as uncomplicated dry eye or viral conjunctivitis may expose patients to the neurological and respiratory adverse effects reported herein without clear therapeutic benefit. This principle of prudent prescribing applies not only to otorhinolaryngology but also to all other clinical settings where these two antihistamines may be used. We therefore hope that this paper may serve as a reference for clinicians in practicing prudent medication use.

Utilizing spontaneous reporting system databases, such as VigiAccess, entails inherent limitations. The variability in the number of reports for each drug, coupled with differing geographical usage patterns and regional habits, can result in a regional skew in adverse reaction reports [40]. Consequently, patterns identified may not be generalizable across different regions. Furthermore, reporting is influenced by regional development levels, with less developed areas potentially lacking the infrastructure and awareness necessary for comprehensive reporting, leading to potential data omissions. In certain instances, the missing data precludes demographic attribution. Additionally, owing to its cumulative data structure, the VigiAccess database does not provide annual ADR breakdowns, leading to variations in the number of collected ADRs for drugs marketed at different times and complicating direct comparisons of signaling profiles. Thus, extensive data mining may not be feasible. To mitigate this, this study collected data on ADRs and PTs for both drugs up to August 31, 2024. The findings of this study were limited to the relative outcomes of the two antihistamines, azelastine and levocabastine.

## Conclusion

Antihistamines play a crucial role in the treatment of ARs. Studies have shown that WHO-VigiAccess has reported no less than 10, 000 cases of adverse reactions to both azelastine and levocabastine treatments. These adverse reactions were characterized by respiratory dysfunction and neurological abnormalities, consistent with previous studies confirming symptoms such as respiratory burning sensation, mucosal dryness, sedation, and vertigo. In addition, levocabastine, which is also an NTS2 antagonist, was associated with more prominent adverse effects than azelastine in terms of neurologic dysfunction. Although serious AEs accounted for only a small proportion of reports for both drugs, this finding should not be overlooked. Conducting safety studies of pharmaceutical products, including cohort event monitoring, is essential for governments to determine the causal association between adverse responses and pharmaceuticals. These results might also be made publicly available through open access repositories to raise awareness of side effects connected to biotechnology-based medications.

## Supporting information

S1 Table Same ADRs between two antihistamines. (DOCX)

S2 Table Different ADRs between two antihistamines. (DOCX)

## References

[1] Settipane RJ, Hagy GW, Settipane GA. Long-term risk factors for developing asthma and allergic rhinitis: a 23-year follow-up study of college students. Allergy Proc. 1994;15(1):21–5. doi: 10.2500/108854194778816634 8005452

[2] Rosario Filho NA, Satoris RA, Scala WR. Allergic rhinitis aggravated by air pollutants in Latin America: A systematic review. World Allergy Organ J. 2021;14(8):100574. doi: 10.1016/j.waojou.2021.100574 34471459 PMC8387759

[3] Klimek L, Sperl A, Becker S, Mösges R, Tomazic PV. Current therapeutical strategies for allergic rhinitis. Expert Opin Pharmacother. 2019;20(1):83–9. doi: 10.1080/14656566.2018.1543401 30439290

[4] Manole F, Bayar Muluk N, Oğuz O, Ulusoy S, Scadding GK, Prokopakis E, et al. Local allergic rhinitis - a narrative review. Eur Rev Med Pharmacol Sci. 2024;28(3):1077–88. doi: 10.26355/eurrev_202402_35344 38375713

[5] Pu X, Wang X, Chen Y, Wang H, Wang X, Yin J. Application of proteomics in allergic rhinitis. Lin Chuang Er Bi Yan Hou Tou Jing Wai Ke Za Zhi. 2022;36(2):153–7. doi: 10.13201/j.issn.2096-7993.2022.02.017 35172557 PMC10128312

[6] Green RJ, Van Niekerk A, McDonald M, Friedman R, Feldman C, Richards G, et al. Acute allergic rhinitis. S Afr Fam Pract (2004). 2020;62(1):e1–6. doi: 10.4102/safp.v62i1.5154 33054254 PMC8377864

[7] Zhang Y, Lan F, Zhang L. Update on pathomechanisms and treatments in allergic rhinitis. Allergy. 2022;77(11):3309–19. doi: 10.1111/all.15454 35892225

[8] Zhang Y, Lan F, Zhang L. Advances and highlights in allergic rhinitis. Allergy. 2021;76(11):3383–9. doi: 10.1111/all.15044 34379805

[9] Bousquet J, Anto JM, Bachert C, Baiardini I, Bosnic-Anticevich S, Walter Canonica G, et al. Allergic rhinitis. Nat Rev Dis Primers. 2020;6(1):95. doi: 10.1038/s41572-020-00227-0 33273461

[10] Ponda P, Carr T, Rank MA, Bousquet J. Nonallergic rhinitis, allergic rhinitis, and immunotherapy: advances in the last decade. J Allergy Clin Immunol Pract. 2023;11(1):35–42. 36152989 10.1016/j.jaip.2022.09.010

[11] Czech EJ, Overholser A, Schultz P. Allergic Rhinitis. Med Clin North Am. 2024;108(4):609–28. 38816106 10.1016/j.mcna.2023.08.013

[12] Meng Y, Wang C, Zhang L. Advances and novel developments in allergic rhinitis. Allergy. 2020;75(12):3069–76. doi: 10.1111/all.14586 32901931

[13] Steelant B, Seys SF, Van Gerven L, Van Woensel M, Farré R, Wawrzyniak P, et al. Histamine and T helper cytokine-driven epithelial barrier dysfunction in allergic rhinitis. J Allergy Clin Immunol. 2018;141(3):951–963.e8. doi: 10.1016/j.jaci.2017.08.039 29074456

[14] Wise SK, Damask C, Greenhawt M, Oppenheimer J, Roland LT, Shaker MS, et al. A Synopsis of Guidance for Allergic Rhinitis Diagnosis and Management From ICAR 2023. J Allergy Clin Immunol Pract. 2023;11(3):773–96. doi: 10.1016/j.jaip.2023.01.007 36894277

[15] McTavish D, Sorkin EM. Azelastine. A review of its pharmacodynamic and pharmacokinetic properties, and therapeutic potential. Drugs. 1989;38(5):778–800. doi: 10.2165/00003495-198938050-00005 2574665

[16] McNeely W, Wiseman LR. Intranasal azelastine. A review of its efficacy in the management of allergic rhinitis. Drugs. 1998;56(1):91–114. doi: 10.2165/00003495-199856010-00011 9664202

[17] Kaliner MA. Azelastine and olopatadine in the treatment of allergic rhinitis. Ann Allergy Asthma Immunol. 2009;103(5):373–80. doi: 10.1016/S1081-1206(10)60355-9 19927534

[18] Dechant KL, Goa KL. Levocabastine. A review of its pharmacological properties and therapeutic potential as a topical antihistamine in allergic rhinitis and conjunctivitis. Drugs. 1991;41(2):202–24. doi: 10.2165/00003495-199141020-00006 1709851

[19] Noble S, McTavish D. Levocabastine. An update of its pharmacology, clinical efficacy and tolerability in the topical treatment of allergic rhinitis and conjunctivitis. Drugs. 1995;50(6):1032–49. doi: 10.2165/00003495-199550060-00009 8612470

[20] Bernstein JA, Bernstein JS, Makol R, Ward S. Allergic Rhinitis: A Review. JAMA. 2024;331(10):866–77. 38470381 10.1001/jama.2024.0530

[21] Hazell L, Shakir SAW. Under-reporting of adverse drug reactions: a systematic review. Drug Saf. 2006;29(5):385–96. doi: 10.2165/00002018-200629050-00003 16689555

[22] Srba J, Descikova V, Vlcek J. Adverse drug reactions: analysis of spontaneous reporting system in Europe in 2007-2009. Eur J Clin Pharmacol. 2012;68(7):1057–63. doi: 10.1007/s00228-012-1219-4 22294060

[23] Watson S, Chandler RE, Taavola H, Härmark L, Grundmark B, Zekarias A, et al. Safety Concerns Reported by Patients Identified in a Collaborative Signal Detection Workshop using VigiBase: Results and Reflections from Lareb and Uppsala Monitoring Centre. Drug Saf. 2018;41(2):203–12. doi: 10.1007/s40264-017-0594-2 28933055 PMC5808049

[24] González-Mendoza T, Bedolla-Barajas M, Bedolla-Pulido TR, Morales-Romero J, Pulido-Guillén NA, Lerma-Partida S, et al. The prevalence of allergic rhinitis and atopic dermatitis in late adolescents differs according to their gender. Rev Alerg Mex. 2019;66(2):147–53. doi: 10.29262/ram.v66i2.521 31200413

[25] De Martinis M, Sirufo MM, Suppa M, Di Silvestre D, Ginaldi L. Sex and Gender Aspects for Patient Stratification in Allergy Prevention and Treatment. Int J Mol Sci. 2020;21(4):1535. doi: 10.3390/ijms21041535 32102344 PMC7073150

[26] Rosário CS, Cardozo CA, Neto HJC, Filho NAR. Do gender and puberty influence allergic diseases? Allergol Immunopathol (Madr). 2021;49(2):122–5. 33641303 10.15586/aei.v49i2.49

[27] Leth-Møller KB, Skaaby T, Linneberg A. Allergic rhinitis and allergic sensitisation are still increasing among Danish adults. Allergy. 2020;75(3):660–8. doi: 10.1111/all.14046 31512253

[28] Hong S-N, Won JY, Nam E-C, Kim TS, Ryu Y-J, Kwon J-W, et al. Clinical Manifestations of Allergic Rhinitis by Age and Gender: A 12-Year Single-Center Study. Ann Otol Rhinol Laryngol. 2020;129(9):910–7. doi: 10.1177/0003489420921197 32425054

[29] Larenas-Linnemann DES, Antolín-Amérigo D, Parisi C. National clinical practice guidelines for allergen immunotherapy: An international assessment applying AGREE-II. Allergy. 2018;73(3):664–72. 28940450 10.1111/all.13316

[30] Xu C, Zhao H, Song Y, Zhou J, Wu T, Qiu J, et al. The Association between Allergic Rhinitis and COVID-19: A Systematic Review and Meta-Analysis. Int J Clin Pract. 2022;2022:6510332. doi: 10.1155/2022/6510332 36249911 PMC9534623

[31] Oh J, Lee M, Kim M, Kim HJ, Lee SW, Rhee SY, et al. Incident allergic diseases in post-COVID-19 condition: multinational cohort studies from South Korea, Japan and the UK. Nat Commun. 2024;15(1):2830. doi: 10.1038/s41467-024-47176-w 38565542 PMC10987608

[32] Snidvongs K, Seresirikachorn K, Khattiyawittayakun L, Chitsuthipakorn W. Sedative Effects of Levocetirizine: A Systematic Review and Meta-Analysis of Randomized Controlled Studies. Drugs. 2017;77(2):175–86. doi: 10.1007/s40265-016-0682-0 28070872

[33] Uemichi Y, Yasuda S, Mabuchi M, Nakao S, Nagano S, Shimizu T. Effects of Azelastine on Glioblastoma Cells. Anticancer Res. 2025;45(7):2763–71. doi: 10.21873/anticanres.17645 40578949

[34] Lee C, Corren J. Review of azelastine nasal spray in the treatment of allergic and non-allergic rhinitis. Expert Opin Pharmacother. 2007;8(5):701–9. doi: 10.1517/14656566.8.5.701 17376024

[35] Kakiuchi M, Ohashi T, Tanaka K, Ohara N, Kamiyama K, Morikawa K, et al. Central nervous system effects of the novel antiallergic agent HSR-609 and typical antiallergic agents using behavioral and electroencephalographic analyses in dogs. Nihon Shinkei Seishin Yakurigaku Zasshi. 1998;18(5):189–99. 10028490

[36] Lores-Arnaiz S, Karadayian AG, Gutnisky A, Miranda J, Rodríguez de Lores Arnaiz G. Changes in synaptic proteins of the complex PSD-95/NMDA receptor/nNOS and mitochondrial dysfunction after levocabastine treatment. Neurochem Int. 2021;148:105100. doi: 10.1016/j.neuint.2021.105100 34139299

[37] Lores-Arnaiz S, Karadayian AG, Gutnisky A, Rodríguez de Lores Arnaiz G. The low affinity neurotensin receptor antagonist levocabastine impairs brain nitric oxide synthesis and mitochondrial function by independent mechanisms. J Neurochem. 2017;143(6):684–96. doi: 10.1111/jnc.14232 28975622

[38] Gutnisky A, López Ordieres MG, Rodríguez de Lores Arnaiz G. The administration of levocabastine, a NTS2 receptor antagonist, modifies Na(+), K(+)-ATPase properties. Neurochem Res. 2016;41(6):1274–80. 26738992 10.1007/s11064-015-1823-7

[39] Coppola T, Béraud-Dufour S, Antoine A, Vincent J-P, Mazella J. Neurotensin protects pancreatic beta cells from apoptosis. Int J Biochem Cell Biol. 2008;40(10):2296–302. doi: 10.1016/j.biocel.2008.03.015 18456542

[40] Faillie J-L. Case-non-case studies: Principle, methods, bias and interpretation. Therapie. 2019;74(2):225–32. doi: 10.1016/j.therap.2019.01.006 30773344

