# Supplementary material for: Characterization of adverse reactions to two antihistamine drugs: A descriptive analysis from WHO-VigiAccess
Source: PLoS One. 2026 Aug 12;21(8):e0355852. doi: 10.1371/journal.pone.0355852 (PMC13466011; doi:10.1371/journal.pone.0355852)
Supplement: Supplementary file 1 [file pone.0355852.s001.docx]

**S1 Table. Same ADRs between two antihistamines.**

| **System organ classes** | **ADRs** | **Signal N** |
| --- | --- | --- |
| **Respiratory, thoracic and mediastinal disorders** | Nasal discomfort, Asthma, Nasal congestion, Throat irritation, Oropharyngeal pain, Dry throat, Wheezing, Dyspnoea, Sneezing, Rhinalgia, Dysphonia, Cough, Rhinorrhoea, Nasal dryness, Epistaxis | 15 |
| **Eye disorders** | Eye inflammation, Dry eye, Visual impairment, Eye swelling, Ocular hyperaemia, Eyelid oedema, Ocular discomfort, Swelling of eyelid, Eye pain, Vision blurred, Eye irritation, Eye disorder, Eye pruritus, Lacrimation increased | 14 |
| **General disorders and administration site conditions** | Oedema, No adverse event, Drug ineffective, Face oedema, Pyrexia, Therapeutic response decreased, Condition aggravated, Malaise, Asthenia, Pain, Therapeutic product effect incomplete, Application site pain, Fatigue, Feeling abnormal | 14 |
| **Nervous system disorders** | Burning sensation, Hypoaesthesia, Ageusia, Migraine, Paraesthesia, Balance disorder, Headache, Parosmia, Tremor, Sedation, Dysgeusia, Dizziness, Anosmia, Somnolence | 14 |
| **Gastrointestinal disorders** | Dyspepsia, Abdominal pain, Vomiting, Dysphagia, Dry mouth, Paraesthesia oral, Abdominal pain upper, Constipation, Nausea, Swollen tongue, Hypoaesthesia oral, Diarrhoea | 12 |
| **Injury, poisoning and procedural complications** | Product dose omission issue, Off label use, Accidental exposure to product, Product administration error, Wrong technique in product usage process, Product use issue, Product use complaint, Product prescribing error, Product use in unapproved indication | 9 |
| **Skin and subcutaneous tissue disorders** | Hyperhidrosis, Erythema, Dermatitis, Dry skin, Urticaria, Pruritus, Rash, Rash pruritic, Angioedema | 9 |
| **Product issues** | Product packaging quantity issue, Product leakage, Product quality issue, Product container issue, Device issue, Product complaint, Product odour abnormal, Product delivery mechanism issue | 8 |
|  |  |  |
| **Psychiatric disorders** | Insomnia, Listless, Hallucination, Anxiety, Nervousness, Depression, Confusional state, Irritability | 8 |
| **Musculoskeletal and connective tissue disorders** | Myalgia, Arthralgia, Pain in extremity, Muscular weakness, Muscle spasms, Back pain | 6 |
| **Investigations** | Blood pressure increased, Blood glucose increased, Weight increased, Heart rate increased | 4 |
| **Cardiac disorders** | Palpitations, Atrial fibrillation, Tachycardia | 3 |
| **Ear and labyrinth disorders** | Ear discomfort, Ear pain, Vertigo | 3 |
| **Immune system disorders** | Anaphylactic reaction, Drug hypersensitivity, Hypersensitivity | 3 |
| **Infections and infestations** | Conjunctivitis, Sinusitis, Rhinitis | 3 |
| **Renal and urinary disorders** | Urinary retention, Dysuria | 2 |
| **Vascular disorders** | Flushing, Hot flush | 2 |
| **Metabolism and nutrition disorders** | Decreased appetite | 1 |
