# Supplementary material for: Characterization of adverse reactions to two antihistamine drugs: A descriptive analysis from WHO-VigiAccess
Source: PLoS One. 2026 Aug 12;21(8):e0355852. doi: 10.1371/journal.pone.0355852 (PMC13466011; doi:10.1371/journal.pone.0355852)
Supplement: Supplementary file 2 [file pone.0355852.s002.docx]

**S2 Table. Different ADRs between two antihistamines.**

|  | **Azelastine** | **Levocabastine** |
| --- | --- | --- |
| **Blood and lymphatic system disorders** |  | Lymphadenopathy |
| **Cardiac disorders** |  | Cardiac disorder, Cardiac failure, Angina pectoris, Cardiovascular disorder, Arrhythmia |
| **Congenital, familial and genetic disorders** |  | Colour blindness |
| **Ear and labyrinth disorders** | Tinnitus | Ear disorder |
| **Eye disorders** | Erythema of eyelid, Periorbital swelling | Blepharitis, Periorbital oedema, Conjunctival haemorrhage, Eye discharge, Photophobia, Asthenopia |
| **Gastrointestinal disorders** | Retching, Abdominal discomfort, Gastrooesophageal reflux disease, Flatulence, Oral discomfort, Abdominal distension | Lip swelling, Coeliac disease, Colitis microscopic, Glossitis, Breath odour, Haematemesis, Gastrointestinal disorder, Aphthous ulcer |
| **General disorders and administration site conditions** | Treatment failure, Chest discomfort, Chest pain, Drug intolerance, Swelling, Drug ineffective for unapproved indication | Therapeutic response unexpected, Adverse event, Therapeutic product effect decreased, Swelling face, Chills, Application site reaction |
| **Hepatobiliary disorders** |  | Hepatic function abnormal |
| **Immune system disorders** |  | Cross sensitivity reaction, Anaphylactic shock, Anaphylactoid reaction, Allergy to chemicals, Reaction to excipient, Decreased immune responsiveness |
| **Infections and infestations** | Bronchitis, Pharyngitis | Herpes virus infection, Rash pustular, Hordeolum, Trachoma, Infection, Nasopharyngitis, Eye infection, Gingivitis, Furuncle |
| **Injury, poisoning and procedural complications** | Incorrect dose administered, Expired product administered, Incorrect route of product administration, Extra dose administered, Inappropriate schedule of product administration | Exposure during pregnancy, Thermal burns of eye, Arthropod sting, Exposure via skin contact, Wrong product administered, Product storage error, Multiple use of single-use product, Medication error, Accidental overdose, Chemical burns of eye, Accidental exposure to product by child |
| **Investigations** | Weight decreased, Hepatic enzyme increased | Colour vision tests abnormal red-green, Blood pressure decreased, Heart rate irregular |
| **Metabolism and nutrition disorders** | Increased appetite, Polydipsia | Hyperglycaemia, Fluid retention |
| **Musculoskeletal and connective tissue disorders** |  | Sjogren’s syndrome, Musculoskeletal stiffness, Myopathy, Muscle twitching |
| **Neoplasms benign, malignant and unspecified (incl cysts and polyps)** |  | Chronic lymphocytic leukaemia |
| **Nervous system disorders** | Taste disorder, Lethargy, Seizure, Loss of consciousness, Head discomfort, Hypotonia | Disturbance in attention, Brain fog, Burning sensation mucosal, Dreamy state, Dystonia, Facial paralysis |
| **Pregnancy, puerperium and perinatal conditions** |  | Abortion, Unintended pregnancy, Large for dates baby |
| **Product issues** | Device leakage, Product packaging issue, Device defective, Device malfunction, Device breakage, Product after taste, Product taste abnormal, Product substitution issue, Device delivery system issue | Product physical issue, Product availability issue, Product compounding quality issue, Product supply issue |
| **Psychiatric disorders** | Disorientation, Nightmare, Sleep disorder | Aggression, Abnormal dreams, Affect lability, Suicide attempt, Dependence, Depressed mood, Abnormal behaviour, Flat affect, Agitation, Drug dependence, Anger, Mood altered |
| **Renal and urinary disorders** | Pollakiuria | Micturition disorder, Renal impairment |
| **Respiratory, thoracic and mediastinal disorders** | Hiccups, Oropharyngeal discomfort, Nasal pruritus, Sinus disorder, Upper-airway cough syndrome | Bronchospasm, Nasal oedema, Paranasal sinus discomfort, Increased bronchial secretion, Nasal obstruction |
| **Skin and subcutaneous tissue disorders** | Skin exfoliation, Alopecia | Night sweats, Rash erythematous, Dermatitis contact, Dermatitis bullous, Erythema multiforme, Blister, Rash macular, Photosensitivity reaction, Madarosis, Drug reaction with eosinophilia and systemic symptoms, Skin irritation |
| **Social circumstances** |  | Impaired driving ability, Loss of personal independence in daily activities, Bereavement |
| **Surgical and medical procedures** |  | Joint stabilisation, Surgery, Therapy interrupted |
| **Vascular disorders** | Hypertension | Hyperaemia, Peripheral vascular disorder, Circulatory collapse, Raynaud’s phenomenon, Pallor |
