# Supplementary material for: Characterization of adverse reactions to two antihistamine drugs: A descriptive analysis from WHO-VigiAccess
Source: PLoS One. 2026 Aug 12;21(8):e0355852. doi: 10.1371/journal.pone.0355852 (PMC13466011; doi:10.1371/journal.pone.0355852)
Supplement: Supplementary file 3 [file pone.0355852.s004.docx]

Dear Editor and Reviewers:

We sincerely thank you for your time, effort, and insightful feedback on our manuscript. The constructive comments provided have been invaluable in improving the clarity and overall quality of our work.

In response, we have carefully revised the manuscript based on the reviewers' suggestions and have made every effort to minimize grammatical errors. To facilitate the review process, we are providing a clean version of the manuscript as well as a marked version with all track changes. Additionally, a detailed, point-by-point response to each comment has been included.

We are deeply appreciative of the meticulous attention given to our manuscript and sincerely hope that the revised version now fulfills the criteria for publication.

Reviewer #1: Ophthalmologists often come across allergic clinical entities such as vernal keratoconjunctivitis, atopic conjunctivitis and seasonal conjunctivitis; it becomes imperative to prescribe topical antihistamine eyedrops in such patients and the given article appropriately highlights the ADRs of these medications. It is to be emphasized that the use of these eyedrops be limited to the above-mentioned conditions. Keeping in line with the ADRs highlighted in the study, they should be avoided as a blanket therapy in uncomplicated cases of dry eye syndrome and viral conjunctivitis - two very commonly encountered conditions where antihistamines are often indiscriminately prescribed by clinicians.

**Response:**

We are very grateful for the reviewer’s positive evaluation of our manuscript, and we are truly honored by your recognition. We also sincerely thank the reviewer for the insightful clinical perspectives shared from the viewpoint of ophthalmic indications. Although this paper primarily focuses on the use of antihistamines in allergic rhinitis, the reviewer highlighted that indiscriminate use in non-allergic conditions does occur in clinical practice. Accordingly, we have added this point to the Discussion section, hoping to provide a reference for ophthalmologists in clinical practice. (Line 371-378, manuscript)

Reviewer #2: The manuscript provides a clear and concise overview of spontaneously reported AEs for 2 antihistamine drugs. Several comments and suggestions have been identified which should be considered by the authors.

**Response:**

We sincerely thank the reviewer for the thorough and insightful review of our manuscript. These detailed comments have substantially enhanced the rigor, transparency, and readability of this paper. We have fully addressed the major concerns by clarifying the screening criteria for ICSRs, defining the reference group used for the disproportionality analysis, and reorganizing the Discussion section to highlight the main findings and compare the two drugs. In addition, we have implemented all other suggestions regarding terminology, figure and table presentation, and methodological transparency. These revisions have markedly improved the quality of the manuscript.

- The pathogenesis of AR is clearly described. To improve readability, consider to include a figure for the pathogenesis.

**Response:**

We greatly appreciate your positive evaluation and careful reading of our manuscript. In response to your suggestion, we have added a figure illustrating the pathogenesis of allergic rhinitis in the Introduction, to provide a visual overview of the key pathophysiological processes and enhance the readability of the article. The newly added schematic diagram of the pathogenesis is shown as follows:

Fig 1:

- Lines 67-73: The information in this section is not consistent as both drugs are second generation antihistamines and are selective H1-antagonists. In the current version it is specifically stated that azelastine is a second generation antihistamine and levocabastine is a selective H1-receptor antagonist. As this applies to both antihistamines the authors are advised to amend the manuscript accordingly.

**Response:**

We sincerely thank you for pointing out this inconsistency. We have revised the relevant sections accordingly, and in the revised version, both drugs are clearly identified as second-generation antihistamines and selective H1 receptor antagonists, thereby ensuring consistency throughout the text (Line 68-75, manuscript). The revised text reads as follows:

Azelastine, a phthalazinone derivative, is a second‑generation antihistamine and a selective H1‑receptor antagonist. It exhibits unique pharmacological properties by inhibiting the synthesis and/or expression of various chemical mediators of allergic reactions [15], such as leukotrienes, kallikreins, cytokines, chemokines, and superoxide radicals [16, 17]. Levocabastine is also a second‑generation antihistamine and a selective, long‑acting H1‑receptor antagonist administered via the nasal and ocular routes. In controlled trials, levocabastine has been shown to be both efficacious and well‑tolerated in the treatment of allergic rhinitis and allergic conjunctivitis [18, 19].

- Lines 88-100: This information is better placed in the methods section of the manuscript. Please amend.

**Response:**

We greatly appreciate the reviewer’s suggestion. We have moved this content to the Data Sources subsection of the Methods section, thereby improving the logical flow and structural integrity of the manuscript. (Line 136-146, manuscript)

- Lines 120-124: From the information in the methods section it can not be deduced when ICSRs are selected and excluded. Within spontaneous reports medicines can be either classified by the reporter as suspected or concomitant. Which reports are included in the current study? And are any exclusion criteria applied?

**Response:**

We thank the reviewer for this important comment. We have revised the Methods section to clarify the ICSR selection and exclusion criteria. Specifically, only reports in which azelastine or levocabastine was classified as a "suspected" drug were included in the analysis; reports where these drugs were listed solely as "concomitant" medications were excluded. To ensure data quality, we excluded duplicate reports and reports with incomplete adverse event information. These criteria have now been explicitly stated in the Methods section. (Line 161-167, manuscript)

- Line 128: anti-VEGF drug need to be replaced by antihistamine drugs.

**Response:**

We greatly appreciate your careful reading of our manuscript, which has been immensely helpful in improving the scientific quality and accuracy of our article. We have corrected “anti-VEGF agents” to “antihistamines” (Line 129, manuscript). The revised text reads as follows:

Therefore, a search was conducted of the records for each antihistamine, and individual AEs were identified employing the MedDRA SOC and PT criteria to examine the range of toxicities.

- Disproportionality analysis: Please add which statistical software was used to calculate the ROR and PRR.

**Response:**

We thank the reviewer for this request. In the revised manuscript, we have added a detailed description of the data retrieval and statistical analysis pipeline in the Methods section. Briefly, we accessed the VigiAccess database (https://vigiaccess.org/) using Python 3.10 with the requests library to query all drug BASENAMEs in the WHODRUG dictionary. The JSON data returned from the front-end pages were parsed, converted into structured tables using the Pandas library, and exported to Excel for download. All disproportionality analyses, including the calculation of ROR, PRR, and their 95% confidence intervals, were subsequently performed using SAS 9.4 software. We hope this clarification meets the reviewer’s expectation. (Line 155-160, manuscript)

- Line 137: Shouldn’t 5 drugs be replaced by 2 drugs?

**Response:**

We sincerely thank you for your careful reading of our manuscript, and we apologize for having made such an obvious error. We have now corrected “five drugs” to “two drugs” in the manuscript (Line 149, manuscript). The revised text reads as follows:

This study utilized a retrospective quantitative research design. Descriptive analysis using Excel was conducted to analyze the characteristics of ADR profiles for two drugs.

- Lines 170-173: The authors are advised to include the number of Adverse Events in addition to the number of reports currently included.

**Response:**

We sincerely thank you for your valuable suggestion and have added the number of adverse events to the manuscript (Line 212-214, manuscript). The revised text reads as follows:

The numbers of adverse events covered in these ADR reports were 15,330 for azelastine and 1,292 for levocabastine.

- Lines 175 and 176: Please remove the word significant from these sentences as no statistics are applied.

**Response:**

We sincerely thank you for your valuable suggestion. We have removed the word “significantly” from the sentence (Line 207-210, manuscript). The revised sentence reads as follows:

Table 2 displays, among the 11, 592 reports of these two antihistamines, excluding 972 cases of unknown gender, the number of ADRs reported in females (7, 262, 62.65%) was higher than that of males (3, 376, 29.12%), with a male-to-female ratio of 2.15:1 and a gender difference.

- Table 2: Consider shortening this table by combining age groups and years of reporting.

**Response:**

We sincerely thank you for your valuable suggestion. Since our age groups were classified according to the neonatal period, infancy, childhood, adolescence, adulthood, middle age, and old age, and each age stage is representative to some extent, we have not removed any age groups. However, we fully respect your opinion and recognize that the table was somewhat lengthy; therefore, we have merged the year groups, presenting only the data of the last ten years and combining the data before 2014 into a single group. The revised table is shown below. Should you have any further valuable suggestions, we will certainly give them careful consideration and make revisions to the best of our ability. (Line 226, manuscript, now Table 3)

- Lines 202-210: MAJOR: The authors should specify which AEs were included in the analysis on neurological disorder and from the methods section and the results section it does not become clear which reference group is used for the analysis to calculate the ROR and PRR. Please specify.

**Response:**

We sincerely thank the reviewer for this important comment, which has substantially improved the methodological transparency of our manuscript. In the revised version, we have made the following specific additions to the Methods section:

1. Table 2: We have added a new table summarizing the principles of disproportionality measurement, including the calculation formulas for ROR and PRR, their standard errors, 95% confidence intervals, and the criteria for signal detection.

2. Neurological ADR definition: We have explicitly stated that all PTs belonging to the MedDRA SOC "Nervous system disorders" were included in the neurological disorder analysis. The specific PTs identified in our dataset (including burning sensation, hypoaesthesia, ageusia, migraine, paraesthesia, balance disorder, headache, parosmia, tremor, sedation, dysgeusia, dizziness, anosmia, and somnolence) have been integrated into the Disproportionality analysis subsection of the Methods.

3. Reference group clarification: We have clearly specified that the reference group for all disproportionality analyses consists of all other drug–ADR combinations in the WHO-VigiAccess database. The 2×2 contingency table is constructed with the study drug and target ADR as the index pair (cells *a* and *b*), while all other drugs and all other ADRs in the database serve as the background comparator (cells *c* and *d*). (Line 189-191, Line 195-207, manuscript)

- Table 5: Consider to shorten this table by only presenting the 10 most frequently reported AEs.

**Response:**

We thank the reviewer for the suggestion. In accordance with the suggestion, we have streamlined Table 5 to present only the 10 most frequently reported adverse events. This revision makes the table more concise and enables readers to rapidly identify the safety signals of greatest clinical relevance. The complete list of adverse events remains available upon request. (Line 260, manuscript, now Table 6)

- Figure 1 does not add relevant information, consider to delete.

**Response:**

We thank the reviewer for this suggestion. Upon re-evaluation, we agree that Figure 1 did not provide necessary additional information beyond what was already covered in the main text, and we have therefore removed it from the revised manuscript.

- Table 6 & 7: These tables are quite extensive and have limited added value. Consider to move these tables to the supplementary information.

**Response:**

We thank the reviewer for the suggestion. We agree that Tables 6 and 7 were lengthy and could distract readers from the main findings. Therefore, we have moved these two tables to the supplementary materials (Supplementary S1 and S2 Tables).

- Discussion section: MAJOR: start with a summary of the main findings and discuss the findings in relation to previous studies in the following paragraphs. Please amend. In addition, the information in lines 259-278 is a repetition of the information already described in the introduction section. Please delete.

**Response:**

We sincerely thank the reviewer for the important guidance on improving the Discussion section. We have comprehensively restructured this section accordingly. The revised Discussion opens with a brief summary of the main findings, followed by a paragraph-by-paragraph discussion in which each key result is compared with previous studies. In addition, we have completely removed the repetitive content originally in lines 259–278 and incorporated new research content related to both drugs. The specific revisions are too extensive to be detailed here. (Line 292-307, manuscript)

- From the results section it is interesting to note the difference in AE reports between the two antihistamines. Consider to discuss this difference in the discussion section.

**Response:**

We sincerely thank the reviewer for this insightful suggestion. We agree that the differences in adverse event reporting between the two antihistamines warrant further exploration. We have comprehensively restructured the Discussion section and added a mechanistic discussion of the adverse reactions of both drugs based on literature from recent years. Further details can be found in the Discussion section. (Line 308-370, manuscript)

- Lines 304-319: Please shorten this section and try to avoid repetition of the information already included in the results section.

**Response:**

We thank the reviewer for this suggestion. In accordance with your comment, we have deleted the paragraph in lines 304–319 of the original manuscript and replaced it with a combined analysis of the main results and previous studies. (Line 308-370, manuscript)

- Lines 364-365: Consider to replace this specific information to the results section.

**Response:**

We thank the reviewer for this suggestion. We agree that the content originally in lines 364–365 is more appropriately placed in the Results section, as this information directly pertains to the study findings rather than to the discussion. We have moved this content to the relevant subsection in the Results section to ensure a smoother narrative and better alignment with the standard manuscript structure. The corresponding sentence in the Discussion section has been revised to avoid repetition. (Line 400-402, manuscript)

- Line 365: It is not clear why the term biologics is introduced as both drugs studied are not biologics.

**Response:**

We sincerely thank the reviewer for pointing out this inaccuracy. We acknowledge that azelastine and levocabastine are small-molecule antihistamines, not biologics. The use of the term “biologics” in the original manuscript was a terminological error and has been corrected (Line402-404, manuscript). The revised sentence reads as follows:

Conducting safety studies of pharmaceutical products, including cohort event monitoring, is essential for governments to determine the causal association between adverse responses and pharmaceuticals.
